# Treatment of Oroantral Communication Using the Lateral Palatal Sliding Flap Technique

**DOI:** 10.1155/2015/730623

**Published:** 2015-05-31

**Authors:** Fernando Salimon Ribeiro, Cassio Torres de Toledo, Michele Romero Aleixo, Maria Cristina Durigan, Willian Corrêa da Silva, Samanta Kelen Bueno, Ana Emília Farias Pontes

**Affiliations:** ^1^School of Dentistry, Educational Foundation of Barretos, UNIFEB, Avenue Prof. Roberto Frade Monte 389, Bairro Aeroporto, 14783-226 Barretos, SP, Brazil; ^2^13th Mechanized Cavalry Regiment, Avenue Nilton Prado 2251, Bairro Centro, 13631-900 Pirassununga, SP, Brazil

## Abstract

Herein, we present a case of oroantral communication that was to be treated with clinical examination, tomography, and prototyping. A patient presented with oroantral communication with purulent exudation for 4 months, since the displacement of the dental implant and O-ring component to the maxillary sinus. Tomographic examination and prototyping revealed a 5 mm bone gap. The patient underwent local washes and antibiotic therapy. After local palpation, a bone defect detected by prototyping was suspected to be greater than that observed. For the surgery, a communication tunnel was made, and the bone defect was found to be 12 mm in diameter. A pedicle flap was raised on the palate, followed by sliding and suturing. No complications were observed during the postoperative period, and the suture was removed after a week. Four months later, communication did not resume, and the patient did not complain of pain, foul smelling, or purulent discharge and was satisfied with the outcome. The findings of this case suggest that the lateral sliding flap can be used as an efficient technique for closing oroantral communications. An accurate clinical examination is a critical tool that can be used instead of tomography and prototyping, which can be misleading.

## 1. Introduction

Oroantral communications are pathologic open connections between the oral cavity and maxillary sinus and are relatively frequent complications observed in dentistry. The highest prevalence is observed in men, approximately 40 years of age, and complications are normally observed after dental extraction surgery, where the third molars are at a greater risk [1, 2].

Spontaneous closure of defects, smaller than 3 mm in diameter, can occur; however, larger communications require surgical interventions [3]. Several surgical techniques can be employed, including slide-in flaps [4], use of pedicled or unpedicled buccal fat pads [5, 6], membranes, autogenous, allogeneic, and xenogeneic grafts, and hemostatic agents [3, 7–9].

Scattarella et al. [3] used an autologous bone graft integrated with xenologous particulate bone graft, which was covered with a nonreabsorbable, expandable, polytetrafluoroethylene titanium-reinforced membrane. This guided-tissue regeneration (GTR) technique was successfully employed to optimize the reconstruction of bone tissue and prevent epithelial migration to the grafted area. Regenerative techniques have better prognosis in cases where resorption of the alveolar wall is low, by providing sufficient bone structuring for creation of a space for tissue regeneration and stabilization of the blood clot [10].

Sandhya et al. [7] described a case series of patients with oroantral defects due to dental extractions that were treated with a sandwich graft technique. To prepare the sandwich, a freeze dried mineralized bone allograft was sandwiched in a collagen membrane, which was closed and sutured with a resorbable suture. The sandwich was inserted into the remaining dental alveoli and the remaining granulated graft. Successful closure of the communication was observed during the follow-up period. Hariram et al. [8] performed a comparative study between buccal fat pad grafts and sandwich grafts; the latter was composed of hydroxyapatite crystals embedded within a collagen sheath and sutured with a polyglactin 910 suture. The findings suggested that the sandwich technique provided a better closure of the oroantral communication due to the formation of a layer on the floor of the maxillary sinus that was considered to be suitable for bone regeneration.

In an experimental study by Muglali et al. [9], surgery created acute oroantral communications with a 2 mm bur in extraction sockets of rabbits after premolar extraction. The authors evaluated bone formation using hemostatic agents, comparing the oxidized regenerated cellulose with bone wax or negative controls without graft. The authors observed that both biomaterials did not contribute to the formation of fibrous connective tissue and bone in the oroantral region, which may have been influenced by the small size of the lesion studied.

In dental implants, cases of implant displacement into the sinus after installation of immediate prostheses have been reported in the literature, but only a few cases lead to oroantral communications [11]. Some factors may be responsible for the displacement of the implant into the maxillary sinus, such as low bone quality and bone resorption after surgery, causing mucosal thickening and sinusitis, and potentially lead to chronic infection associated with communication between the oral cavity and maxillary sinus.

This study aimed to present a case of oroantral communication, due to the displacement of a dental implant and O-ring component into the maxillary sinus, which was examined via clinical examination, tomography, and prototyping.

## 2. Case Presentation

A 60-year-old woman with oroantral communication presented for treatment at the Specialization Course in Implant Dentistry of the University Center of the Barretos Educational Foundation (UNIFEB).

The main complaint was foul smelling, purulent discharge and intense facial pain, for which she continued to take analgesic ketorolac trometamol (10 mg). The patient reported to be in good general health, not having undergone radiation therapy, or consumed drugs, alcohol, or tobacco. On clinical examination, total edentulism was observed.

The patient had undergone maxillary sinus lift surgery, and six months later, 6 implants were implanted in the maxilla. Less than a month later, the patient realized that both the implant and prosthetic component installed in the region of tooth 16 had dislocated and was housed within the maxillary sinus. Oroantral communication was the result. A surgical procedure was performed by an otorhinolaryngologist, which only resulted in the removal of the implanted body. Some days later, the patient reported that the prosthetic component was expelled through the nose. However, the communication and local infection persisted, and four months later, the patient sought treatment.

**Figure 1:**
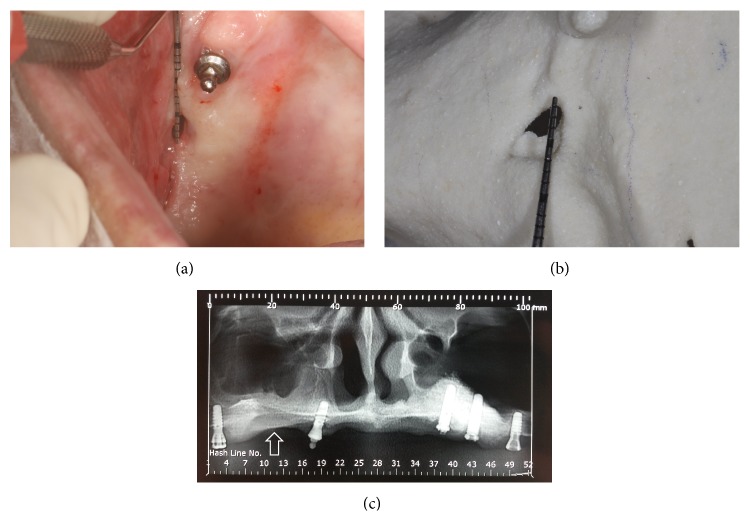
Initial appearance of the patient's condition upon (a) inspection and (b) prototyping, the arrow indicates an 5 mm opening, and (c) radiographic appearance, the arrow indicates oroantral communication.

On initial inspection (Figure 1), the presence of a 5 mm diameter communication was confirmed, with mild mucosal inflammation of the surrounding region. Tomographic examination was requested along with prototyping of the jaw, and a 5 mm bone gap was detected. The treatment plan involved the remission of infection, followed by surgical closure of the injury. The patient was instructed to wash with saline and 0.12% chlorhexidine in a ratio of 1 : 1 using a hypodermic needle attached to a sterile rubber hose without cutting the tip. Furthermore, amoxicillin-clavulanic acid (875 mg) and metronidazole (200 mg) were prescribed for 14 days. After this period, there was no pain, and the local secretion became transparent. However, local palpation revealed that the suspected bone defect was greater than that shown on prototyping.

### 2.1. Surgical Procedure

**Figure 2:**
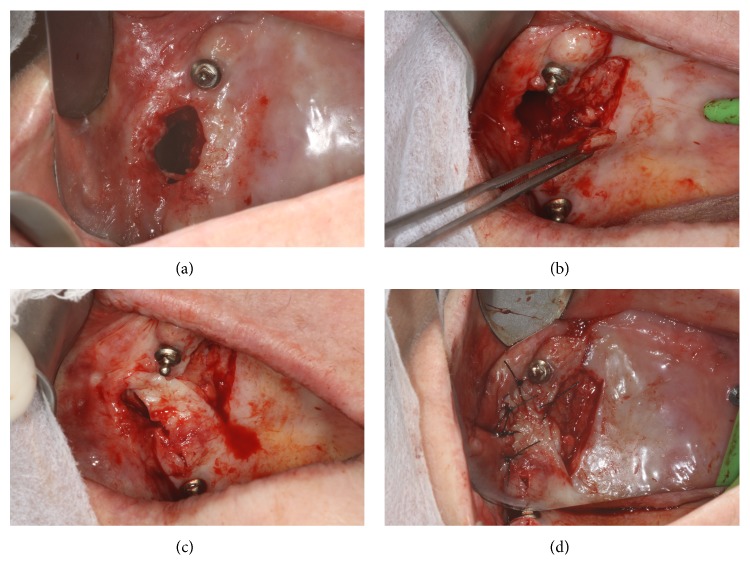
During the surgical procedure (a) an incision was made to allow for the removal of the soft tissue collar. The bone lesion was 12 mm in diameter. A pedicle flap was (b) elevated, (c) laterally slid to cover the defect without tension, and (d) sutured while maintaining a bare surgical bed covered by the periosteum and a thin layer of connective tissue.

On the day of surgery, the patient underwent extrabuccal antisepsis with povidone iodine solution and intrabuccal antisepsis with chlorhexidine 0.12%. The patient underwent infiltration anesthesia around the communication and palate using 4% articaine chloride and epinephrine 1 : 100,000. An incision was made around the communication in order to remove the epithelialized soft tissue collar (Figure 2(a)). At this time, the actual size of the bone lesion was measured at 12 mm at its largest diameter (mesiodistal), which was not consistent with the prototype examination.

A mucosal pedicled flap was elevated (Figure 2(b)) and slid sideways from the palate to cover the communication (Figure 2(c)). Subsequently, local compression was performed for 2 min, followed by suturing with nylon 5.0 simple sutures (Figure 2(d)). The residual denuded area was covered with surgical cement.

After surgery, the patient received instructions on postoperative care; she was instructed not to brush the operated area. The following procedures were prescribed: intrabuccal mouthwash without rinsing with 0.12% chlorhexidine digluconate solution for two weeks, amoxicillin and clavulanic acid 875 mg given orally every 8 h for 7 days, nimesulide 100 mg 12 in 12 h for 3 days, paracetamol 750 mg 6 in 6 h for 3 days, and nasal decongestant oxymetazoline hydrochloride 0.05% every 12 h for 3 days.

**Figure 3:**
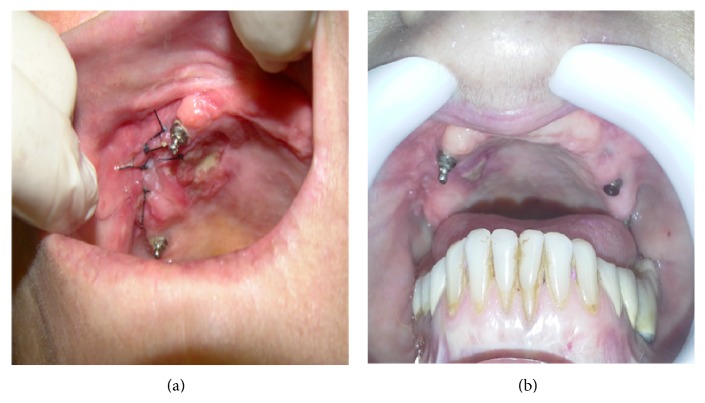
Clinical aspect (a) at one week and (b) one month after the surgery for communication closure.

There were no intercurrences in the postoperative period, and a week later the sutures were removed. After 4 months of observation (Figure 3), the communication remained closed, no pain, foul smelling, or purulent discharge was observed, and the patient was satisfied with the treatment outcome.

## 3. Discussion

This report describes treatment of an oroantral communication due to a displacement of an implant and prosthetic component into the maxillary sinus. The sliding flap technique was successful, enabling communication closure and ensuring patient's satisfaction. The clinical implication is that this technique can be adopted for closure of oroantral fistulas, especially in cases that require keratinized tissue formation over the drilled area, without moving the mucogingival line in the coronal direction, which can facilitate future prosthetic restorations.

During the selection of flap type, the relevance or nonrelevance of vestibular floor reduction must be considered. The sliding of the pedicle flap of the alveolar mucosa and/or buccal mucosa to move the buccal fat pad can make the palate less favorable for stabilization of mucosupported prostheses and reduce the keratinized mucosal band in place, hindering the resolution of cases with aesthetic demand. Reversion of this situation implies the need for an additional surgical procedure [12]. In the present case, we decided to move the palatal flap without undermining the vestibular floor depth and avoid coronal displacement of the mucogingival line. Another advantage of the sliding flap technique is the vascularization of the graft, which is pedicled; however, there are certain disadvantages, such as technique difficulty and morbidity [4]. Nevertheless, prior planning of the location, depth, and direction of the incisions, use of sterile scalpel blades, protection of the donor area with surgical cement stabilized with the suture, and the adequacy of postoperative medication for the needs of individual patients have been used to ensure greater postoperative success.

In the case presented, it was necessary to perform rehabilitation in two phases: first for the remission of sinus infection and second for closure of the communication [4]. The local infection was treated by washing with saline along with oral antibiotics, thereby resulting in remission of the symptoms and signs of microbial contamination, as described by Batra et al. [12].

A surgical procedure was indicated for its closure, because the defect was observed to be >5 mm and also due to persistence of infection for more than 3 weeks [3]. The sliding flap was a simple and effective technique used for the treatment of oroantral communication, which presented no complications during and after surgery. The lesion was sealed efficiently. After four months of surgery, the communication remained closed, with no sign of inflammation and infection, and the patient was satisfied; this period is critical for the postoperative follow-up [13]. Bone grafts or substitutes were not required because the defect was shallow and without sidewalls, which implied a doubtful prognosis for new bone formation, due to lack of stability of the bed. Due to the breadth of the bone defect and soft tissue, we decided not to perform an autogenous block graft, which would reduce the total treatment time [14] as risk was not greater than the benefit.

During surgical planning, computed tomography imaging of the area demonstrated a 5 mm lesion, which was considerably smaller than the 12 mm disclosed during surgery. Comparative studies should be performed with different diagnostic techniques for treatment planning of oroantral communications. Therefore, an accurate clinical examination takes precedence over the use of techniques, such as tomography and prototyping, which can be inaccurate.

Based on the reported findings, it can be concluded that the sliding flap is an efficient technique that can be used for closing oroantral communications.
